# Immuno-related cardio-vascular adverse events associated with immuno-oncological treatments: an under-estimated threat for cancer patients

**DOI:** 10.1007/s00395-024-01077-7

**Published:** 2024-09-03

**Authors:** Giuseppe Panuccio, Pierpaolo Correale, Maria d’Apolito, Luciano Mutti, Rocco Giannicola, Luigi Pirtoli, Antonio Giordano, Demetrio Labate, Sebastiano Macheda, Nicole Carabetta, Youssef S. Abdelwahed, Ulf Landmesser, Pierfrancesco Tassone, Pierosandro Tagliaferri, Salvatore De Rosa, Daniele Torella

**Affiliations:** 1 Department of Cardiology, Angiology and Intensive Care Medicine, Deutsches Herzzentrum der Charité Berlin, 12200 Berlin, Germany; 2 Department of Experimental and Clinical Medicine, Magna Graecia University, Catanzaro, Italy; 3 Medical Oncology Unit, Grande Ospedale Metropolitano Bianchi Melacrino Morelli, 89124 Reggio Calabria, Italy; 4 Sbarro Institute for Cancer Research and Molecular Medicine and Center of Biotechnology, College of Science and Technology, Temple University, Philadelphia, PA 19122 USA; 5 Department of Applied Sciences and Biotechnology, Università dell’Aquila, L’Aquila, Italy; 6 Department of Medical Biotechnology, University of Siena, 53100 Siena, Italy; 7 Unit of Intensive Care Medicine and Anesthesia, Grande Ospedale Metropolitano Bianchi Melacrino Morelli, 89124 Reggio Calabria, Italy; 8 Department of Medical and Surgical Sciences, Magna Graecia University, Catanzaro, Italy; 9 DZHK (German Centre for Cardiovascular Research), 10785 Berlin, Germany; 10 Berlin Institute of Health (BIH), 10178 Berlin, Germany

**Keywords:** CardioOncology, Immunotherapy, Cardioprotection, Cardiovascular prevention, Heart failure, Cancer therapy

## Abstract

Immunotherapy represents an emergent and heterogeneous group of anticancer treatments harnessing the human immune-surveillance system, including immune-checkpoint inhibitor monoclonal antibodies (mAbs), Chimeric Antigen Receptor T Cells (CAR-T) therapy, cancer vaccines and lymphocyte activation gene-3 (LAG-3) therapy. While remarkably effective against several malignancies, these therapies, often in combination with other cancer treatments, have showed unforeseen toxicity, including cardiovascular complications. The occurrence of immuno-mediated adverse (irAEs) events has been progressively reported in the last 10 years. These irAEs present an extended range of severity, from self-limiting to life-threatening conditions. Although recent guidelines in CardioOncology have provided important evidence in managing cancer treatments, they often encompass general approaches. However, a specific focus is required due to the particular etiology, unique risk factors, and associated side effects of immunotherapy. This review aims to deepen the understanding of the prevalence and nature of cardiovascular issues in patients undergoing immunotherapy, offering insights into strategies for risk stratification and management.

## Background

Over recent years, advancements in supportive care and personalized anticancer treatments have significantly improved cancer patients’ prognoses, increasing the number of long-term survivors. Anticancer immunotherapy is a heterogeneous treatment modality based on the use of biological agents to enhance the immune system’s ability to fight tumors [59]. While early approaches often failed, the discovery of primary immune checkpoints led to the development of monoclonal antibodies (mAbs) able to bind and block these natural inhibitory pathways. The inhibitory immune-checkpoints represent physiological mechanisms in the host, that prevent immunological overreactions and that are able to abrogate the host natural immune response in case of chronic inflammatory processes and cancer. Specifically, mAbs targeting the Programmed cell death receptor 1 (PD-1)/PD-ligand 1 (PD-L1) immune checkpoint produce their staggering anticancer activity by restoring the cytolytic activity of specific immune-effectors in the tumors. These immune-effectors are usually represented by CD8^+^ T cells (cytotoxic T cells, CTLs) whose precursors have risen in the host, along the lengthy process of cancerogenesis [46]. Immune-check points, such as CTLA-4 (expressed on activated CTL precursors and that binds B7.1 in the lymph-nodes) and PD-1 (expressed on tumor infiltrating CTLs and which binds PD-L1 and or PD-L2 in the tumor) may, consequently, become opportunistic instruments of the tumor to escape the immuno-surveillance system [96]. Targeting these checkpoints has revolutionized the treatment for several malignancies, including non-small-cell lung cancer (NSCLC), head and neck carcinoma (HNC), urothelial/kidney cancer, malignant melanoma, triple-negative breast cancer (TNBC), and several gastrointestinal and gynecological malignancies. ICIs target specific immune checkpoints, reviving the immune response to target and erase cancer cells [52]. Additionally, cancer vaccines introduce antigens or immune-stimulating agents to prompt the immune system to recognize and attack cancer cells [69]. Finally, adoptive cell transfer therapies like chimeric antigen receptor T (CAR-T) cell therapy genetically modify a patient’s T cells to better recognize and eliminate cancer cells [37]. Leveraging the body’s immune system, these drugs have displayed remarkable efficacy in prolonging progression-free survival (PFS) and overall survival (OS) in clinical trials. However, they also pose risk of adverse events, particularly cardiovascular and hemodynamic disorders (CHDs) [8]. In fact, since their first use, these agents showed potential cardiovascular side effects, including myocarditis, pericardial diseases and arterial thromboembolic events [12, 82, 85]. The European Society of Cardiology (ESC) emphasize the importance of early detection and preventive measures for CHDs in cancer patients. Guidelines recommend multidisciplinary risk evaluations considering factors like age, lifestyle, type of anticancer treatment and existing cardiovascular conditions [49]. Over 70% of long-term cancer survivors develop CHDs, significantly impacting their quality of life and leading to increased medical expenses [71]. The Heart Failure Association (HFA) and the International Cardio-Oncology Society (ICOS) have developed a stratification tool to classify cancer patients into cardiovascular risk groups [7, 48]. The development of innovative molecular target therapy and immune-oncological drugs poses a significant challenge in preventing and managing adverse events, distinct from traditional antiblastic agents. Managing the cardiovascular side effects of innovative cancer treatments, especially when combined with traditional therapies, is challenging [29, 84]. This review examines the cardiovascular implications of various immunotherapy drugs.

## Immune-oncological drugs

Table 1 provides comprehensive data on available immuno-oncological treatments.

**Table 1:** Immuno-oncological treatments and randomized studies reporting CTR-CVT [3, 6, 9, 10, 31, 38, 66, 75, 80, 90]

Treatment	Mechanism of Action	Approved Uses	Study reference	Cancer type	ICI arm	Comparison arm	CTR-CVT type	CTR-CVT incidence (%)
Ipilimumab (Yervoy)	CTLA-4 inhibitor	Advanced melanoma, renal cell carcinoma, metastatic colorectal cancer, hepatocellular carcinoma	Hodi et al., 2010	Melanoma	Ipilimumab plus gp100/Ipilimumab alone	Gp 100	Dyspnea	12.7
Nivolumab (Opdivo)	PD-1 inhibitor	Advanced melanoma, non-small cell lung cancer, renal cell carcinoma, Hodgkin lymphoma, squamous cell carcinoma of the head and neck, urothelial carcinoma, hepatocellular carcinoma	Borghaei et al., 2015	NSCLC	Nivolumab	Docetaxel	Dyspnea, Pericardial effusion	0.7
Pembrolizumab (Keytruda)	PD-1 inhibitor	Advanced melanoma, non-small cell lung cancer, head and neck squamous cell carcinoma, classical Hodgkin lymphoma, urothelial carcinoma, microsatellite instability-high cancer, gastric cancer	Robert et al., 2015	Advanced Melanoma	Pembrolizumab	Ipilimumab	HTN	0.7
Atezolizumab (Tecentriq)	PD-L1 inhibitor	Non-small cell lung cancer, small cell lung cancer, urothelial carcinoma, triple-negative breast cancer	Socinski et al., 2018	NSCLC	Atezolizumab + Bevacizumab + Carboplatin + Paclitaxel	Bevacizumab + NSCLC + Carboplatin + Paclitaxel	MI, HF, HTN	0.5
Durvalumab (Imfinzi)	PD-L1 inhibitor	Non-small cell lung cancer, extensive-stage small cell lung cancer	Antonia et al. (2017)	NSCLC	Durvalumab	Placebo	MI, AF, HF, Pericardial effusion, cardiogenic shock, VT, HTN	3.3
Avelumab (Bavencio)	PD-L1 inhibitor	Merkel cell carcinoma, urothelial carcinoma	Barlesi et al., 2018	NSCLC	Avelumab	Docetaxel	MI, HF, Myocarditis, HTN	1.5
Relatlimab (Opdualag)	LAG-3 inhibitor	Melanoma	Tawbi et al., 2022	Melanoma	Relatlimab + Nivolumab	Nivolumab	Myocarditis	1.7
Axicabtagene ciloleucel (Yescarta)	CAR T-cell therapy	Diffuse large B-cell lymphoma and high-grade B-cell lymphoma	Westin et al., 2023	Diffuse large B-cell lymphoma	Axicabtagene ciloleucel	Two to three cycles of chemoimmunotherapy followed by high-dose chemotherapy with autologous stem-cell transplantation in patients who had a response	Hypotension; CRS; Cardiac arrest (1 patient)	9; CRS 92%;
Tisagenlecleucel (Kymriah)	CAR T-cell therapy	B-cell acute lymphoblastic leukaemia; Diffuse large B-cell lymphoma and follicular lymphoma	Bishop et al., 2022	Diffuse large B-cell lymphoma	Tisagenlecleucel with optional bridging therapy	Salvage chemotherapy and autologous hematopoietic stem-cell transplantation (HSCT)	CRS	CRS 61.3%
Lisocabtagene maraleucel (Breyanzi)	CAR T-cell therapy	Diffuse large B-cell lymphoma; high-grade B-cell lymphoma; Primary mediastinal large B-cell lymphoma; Follicular lymphoma grade 3B	Kamdar et al., 2022	Relapsed or refractory large B-cell lymphoma	Lisocabtagene maraleucel	Three cycles of salvage immunochemotherapy	Hypotension; CRS	22.8 CRS 50%

*NSCLC*, non-small cell lung cancer; *HTN*, hypertension; *MI*, myocardial infarction; *HF*, heart failure; *AF*, atrial fibrillation; *VT*, ventricular tachycardia; *CRS*, cytokine release syndrome

### Cytotoxic T lymphocyte antigen-4 (CTLA-4)

**Fig. 1:**
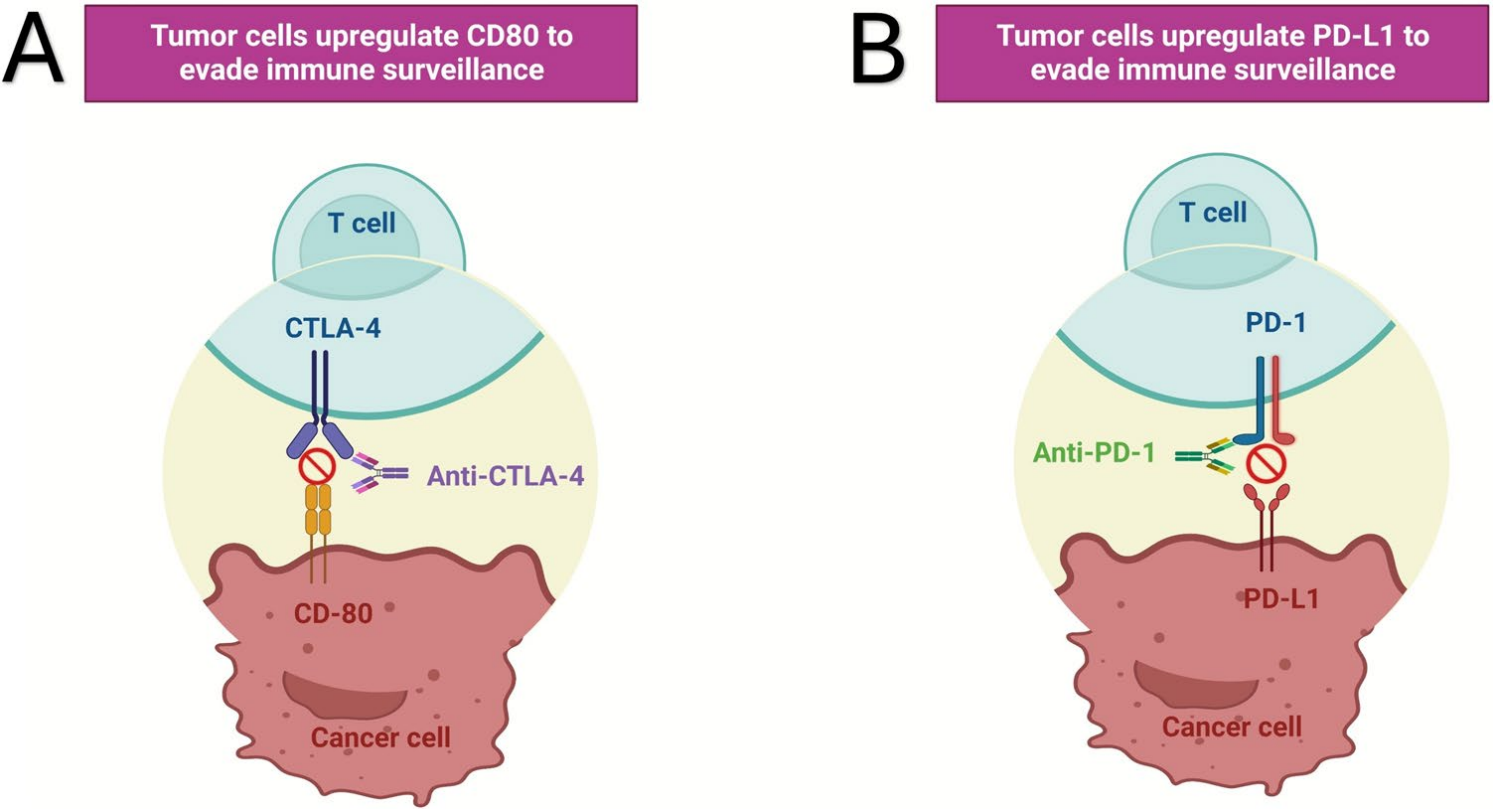
Immunotherapy drugs mechanisms; **A** CTLA-4 mAbs; **B** PD-1/PD-L1 mAbs

CTLA-4 pathway was the first inhibitory immune-checkpoint to be identified. CTLA-4 is a trans-membrane glycoprotein expressed on the surface of activated T cells, competing with CD28 for binding B7.1 and B7.2 on antigen-presenting cells (APCs) and B7H on tumors. CTLA-4 binding sends an inhibitory signal to T cell precursors, that become mostly anergic (Fig. 1A). Ipilimumab and tremelimumab were the first two mAbs against CTLA-4 approved for clinical use for the treatment of malignant melanoma and other malignant [55]. These mAbs restore the proliferative activity of T cell precursors in cancer patients [68]. However, early application of CTLA-4-ICI revealed significant challenges, including several immune-mediated adverse effects. Notably, these adverse events extended to the cardiovascular system, triggering episodes of pericarditis and myocarditis. Ipilimumab-related pericarditis has been reported as a late event, potentially self-limiting or rapidly progressing to cardiac tamponade [88, 92]. The use of CTLA-4 ICI has been sporadically associated to other CHDs including left ventricular dysfunction and myocardial fibrosis whose diagnosis and treatment remains mostly empiric [63, 67, 87]. Massive inflammatory cytokine release syndrome (CRS) may trigger cardiological, hemodynamic and respiratory failure [91]. The assessment of cancer-therapy related cardiovascular toxicity (CTR-CVT) presents challenges due to the differences in how cardiovascular and oncological outcomes are prioritized. Evaluation of CRS represents an example in these disparities. In fact, several scales have been developed to grade the severity of CRS, reflecting different perspectives from cardiology and oncology. More in detail, Lee criteria categorizes CRS based on clinical symptoms, focusing on the presence of fever, organ toxicity and the need for interventions like vasopressors or mechanical ventilation [43]. Penn criteria also include specific cardiovascular parameters such as hypotension and cardiac dysfunction, reflecting a broader assessment including cardiovascular impact [62]. Finally, the American Society for Transplantation and Cellular Therapy (ASTCT) grading for CRS includes clinical and laboratory markers, aligning with oncological practices while considering cardiovascular symptoms [44]. While oncological assessments prioritize managing CRS symptoms to continue cancer therapy, cardiology assessment emphasize detailed cardiovascular evaluation to prevent long-term complications. To close these gaps, multidisciplinary approach is essential. The incidence of irAEs notably increases when CTLA-4 inhibitors are combined with anti-PD-1 mAbs like nivolumab, emphasizing the need for patient monitoring [36]. Furthermore, despite high dosage of ICI could suggest an increased incidence of IrAEs, recent evidence did not show a significant impact of ICI dosage on the occurrence of cardiovascular events [54]. ECG changes, including ventricular arrhythmias, heart blocks, low voltages and pathological Q waves have shown to be related with ICI-related adverse cardiac events [64]. Therefore, ECG monitoring is essential for the surveillance of possible ICI-related cardiovascular toxicity.

### Programmed cell death receptor 1 (PD-1) and PD-ligand 1 (PD-L1) immune checkpoint inhibitor mAbs

The advent of PD-1 and PD-L1 ICIs marked a significant breakthrough in cancer therapy. These inhibitors act on peripheral CTLs, modulating immune responses in chronic inflammation and cancer settings. PD-1, a transmembrane receptor on activated CTLs, binds to PD-L1 and PD-L2 on inflammatory and tumor cells (Fig. 1B). Clinical evaluations have shown that PD-1 and PDL-1 mAbs can reactivate anergic tumor-infiltrating CTLs, restoring robust antitumor activity [79]. However, their use is associated with irAEs, including autoimmune responses, leading to severe cardiac complications like myocarditis and pericarditis. Recent studies have identified a correlation between irAE risk and specific HLA alleles [16]. On these bases, it cannot be excluded a potential link between specific high-risk HLA alleles and the risk of ICI-related cardiovascular events, supported by preclinical studies where genetically engineered mice expressing high-risk HLA-DQ8.abo developed spontaneous autoimmune myocarditis, hinting at regulatory CD4 + T cell dysfunction [77, 86]. Moreover, certain high-risk HLA alleles correlate with irAEs occurrences, including HLA-B35 and HLA-DRB1.11 associated with pneumonitis [78]. PD-1 inhibitors such as nivolumab, pembrolizumab, cemiplimab and dostarlimab, along with PD-L1 inhibitors atezolizumab, avelumab, and durvalumab, have shown variable degrees of cardiotoxicity, including cardiac ischemia and myocardial infarction [56]. Preclinical studies suggest PD-L1’s role in post-ischemic tissue repair, indicating that PD-L1 mAbs might affect these mechanisms, increasing cardiovascular risk [47]. A meta-analysis involving more than 20,000 patients receiving different ICI combinations, particularly double ICI blockade (Ipilimumab and Nivolumab), highlights a significantly elevated risk of irAEs with ICI combinations, particularly, ipilimumab and nivolumab [32]. Moreover, combined PD-1/PD-L1 mAbs with cardiotoxic chemotherapy showed an increased risk of severe cardiac arrhythmias and myocardial damage [73]. IrAEs can also be influenced by the kinetics of mABs. Anti-PD-1 mAbs target PD-1 + T cells directly, while anti-PD-L1 mAbs bind to PD-L1 expressing tissues, including cardiac muscle cells, potentially causing damage [60]. Recent preclinical studies show that PD-L1 is over-expressed in normal tissues during post-inflammatory repair or post-ischemic recovery, exerting protective anti-apoptotic effects. Anti-PD-L1 mAbs may disrupt these properties, increasing the risk of cardiovascular adverse events in patients with preexisting or subclinical ischemic disease [15, 89]. The incidence of ICI-related cardiac adverse events varies widely among tumor entities. More in detail, thymic epithelial tumors, in particular thymoma, are more frequently associated with ICI-related cardiotoxicity [20]. Immuno-related arrhythmias, which pose significant mortality risks, may result from concurrent irAEs or preexisting conditions. These arrhythmias include conduction delays and ventricular and supraventricular arrhythmias, with immune-related arrhythmias involving severe cardiac dysfunction presenting worse prognosis [33]. The occurrence of ICI related auto-immunity can directly affect the cardiac tissues and the cardiovascular system resulting in the occurrence of myocarditis, pericarditis, heart failure, arrhythmias, Tako-Tsubo syndrome (TTS), dyslipidemia, and myocardial infarction (central illustration). Additionally, ICI induced systemic inflammatory syndrome and associated irAEs involving endocrine glands, may indirectly affect heart function, increasing arterial and thromboembolic risk. These findings highlight the importance of baseline evaluations and rigorous cardiac monitoring, especially in patients undergoing PD-1 blockade, chemotherapy, or combined immunotherapy.

### Lymphocyte activation gene-3 (LAG-3) Therapy

**Fig. 2:**
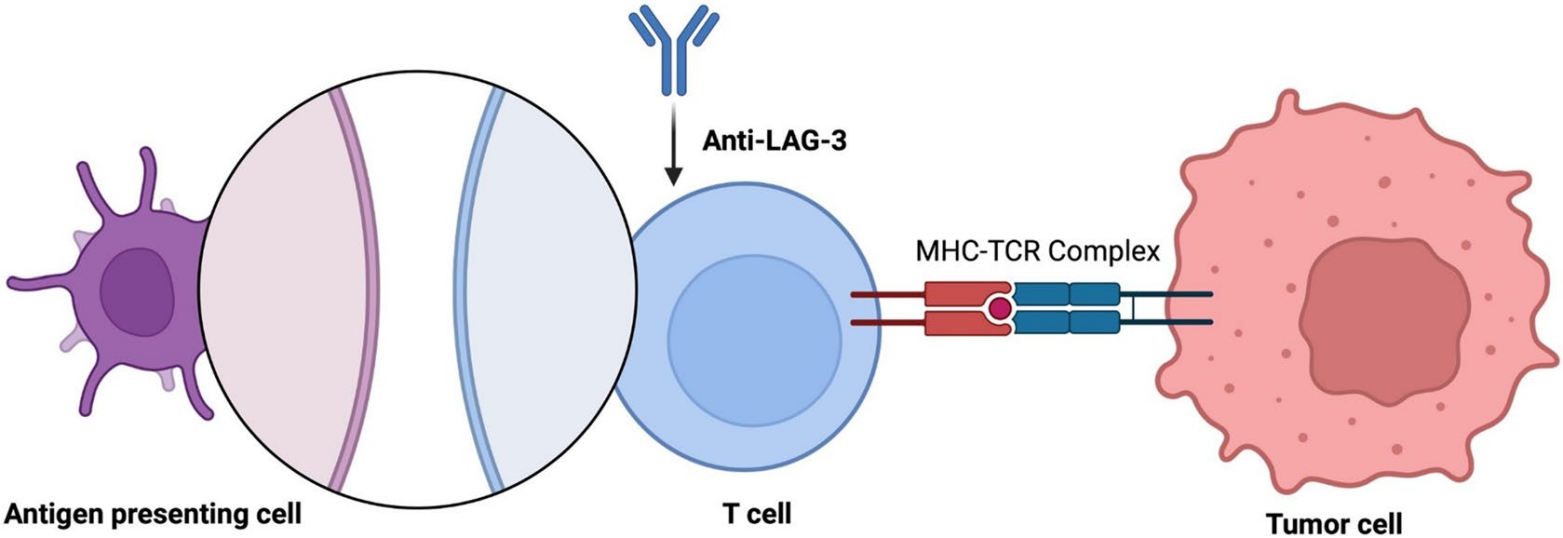
Lymphocyte activation gene-3 (LAG-3) therapy

LAG-3 is an immune checkpoint molecule that regulates both CD4 + and CD8 + T-cell activation. Its primary function is to bind MHC class II molecules, acting as a negative regulator. By reducing T cell activation and proliferation, LAG-3 helps to prevent hyperactive immune responses and promotes immune tolerance [1]. LAG-3’s role in maintaining immunological homeostasis is further highlighted by its co-expression with other immune checkpoints, most notably PD-1, and its interaction with APCs (Fig. 2). Due to these mechanisms, LAG-3 is a therapeutic target for regulating immune responses in various clinical situations, including cancer [18]. Preclinical evidence showed that mice deficient in both LAG-3 and PD-1 developed severe myocarditis, suggesting that LAG-3 acts synergistically with PD-1 to prevent autoimmunity [57]. In a phase 3 study with a LAG-3 inhibitor (Relatlimab) in association with a PD-1 inhibitor (nivolumab), myocarditis occurred in 1.7% of the patients [80].

### Active specific immunotherapy: cancer vaccines

**Fig. 3:**
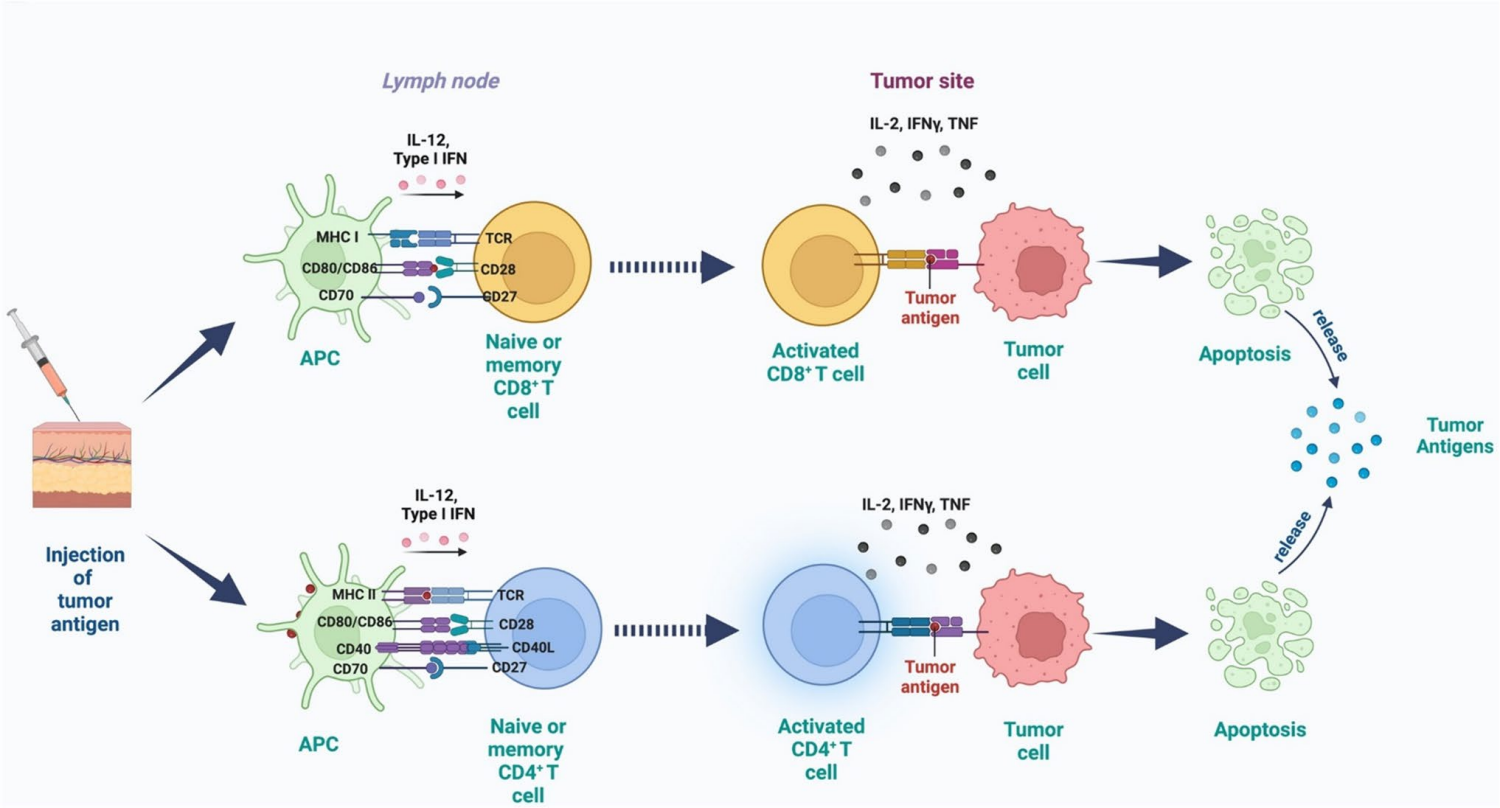
Cancer vaccines injection and pathways generating immune response against cancer cells

Active specific immunotherapy, also known as “cancer vaccines”, operates on the premise that cancer cells express altered genes products known as tumor specific antigens (TSA) and tumor associate antigens (TAA), recognized by sensitized CTLs. These immune cells act as “magic bullets” to identify and kill cancer cells while sparing healthy tissues. Cancer vaccines mimic the natural immunization mechanisms and deliver constructs with target antigens to lymphoid organs, sensitizing specific T cell precursors and triggering an efficient T cell-mediated immune-response (Fig. 3) [72].

Cancer vaccines are classified in: (1) multi-antigen cell-based vaccines; (2) mono or poli-gene engineered viruses-based vaccines, (3) mono or poly-antigen gene engineered nucleic acids-based and finally, (4) peptide/epitope -based vaccines [81]. Currently, no cancer vaccine is approved yet for standard treatment in Europe. The multi-epitope Sipuleucel-T vaccine resulted efficacious in clinical trials and received FDA approval for the treatment of advanced prostate cancer patients over a decade ago, showing immuno-response and safety, and it was rarely associated to autoimmunity or cardiovascular adverse events, including myocardial infarction and thrombo-embolism [30]. In the most recent years, a great impulse to the development of cancer vaccines has followed the recent COVID-19 pandemics [34]. Newly developed nucleic acid (DNA/RNA) and engineered viruses-based vaccines effectively trigger immune-response against tumor antigens, mimicking a viral infection in the host. The first vaccines belonging to this family used viral DNA and RNA backbones (poxvirus, adenovirus, Castle virus, etc.) engineered to express the genes of known tumor target antigens/co-accessory molecules’ (B7.1, ICAM-1 LFA-3, CD40, TRICOM- PSA and TRICOM CEA) and/or proinflammatory cytokines (GM-CSF, IL12). Vaccines like prostate-specific antigen (PSA)-directed vaccine PROSTVAC (Poxoviruses engineered to express the genes of PSA, B7-1, ICAM-1, and LFA-3) showed controversial results in prostate cancer patients [25]. More recently, recombinant mRNA vaccines have been proposed as potential anticancer drugs for colorectal cancer, malignant melanoma, and non-small cell lung cancer [93]. The main safety risk associated with mRNA vaccines is their ability to trigger potent immune reactions and inflammatory syndromes, potentially leading to cardiovascular damage. Cancer vaccines might become a viable therapeutic option for patients with cold tumors, or those refractory to ICIs. However, more clinical trial data are needed to assess the benefits and risks of combining vaccines with ICIs.

### Chimeric antigen receptor T Cells (CAR-T) and cardiovascular damage

**Fig. 4:**
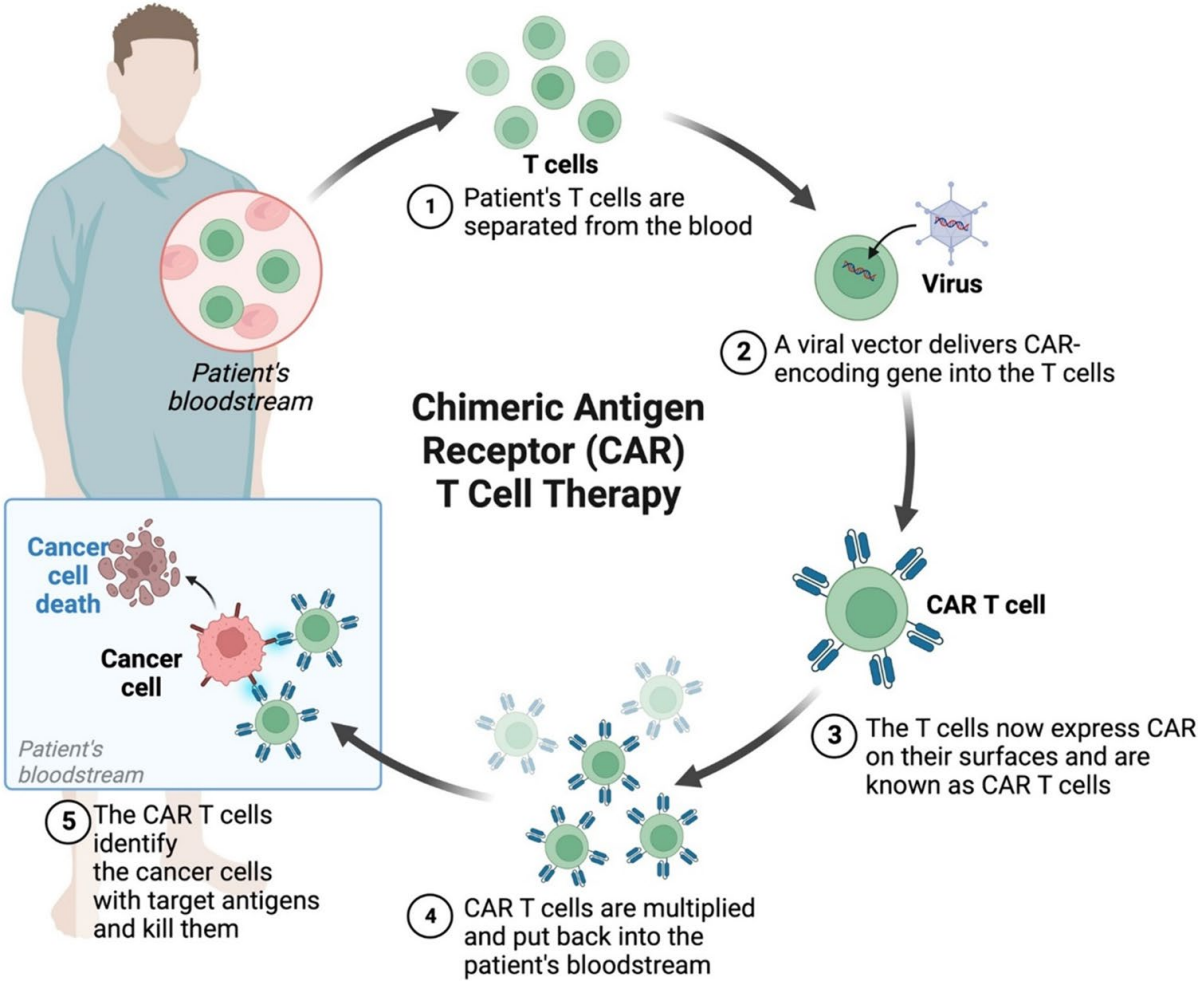
Chimeric antigen receptor (CAR) T cell therapy mechanisms

CAR-T cell therapy has gained prominence in treating selected blood cell malignancies. This approach relies on sensitized antigen-specific CTLs that recognize and eliminate tumors by engaging a trimeric complex involving the T cell receptor and MHC-presented antigens [76]. Despite decades of research into adoptive therapy using autologous antigen-specific CTLs, challenges like ex vivo culture, unpredictable effector homing, and declining cytolytic activity have limited its clinical translation [95]. However, advances in gene engineering have enabled the clinical development of CAR-T therapies, which involve infusing engineered T cells expressing selected TCR genes or other recognition molecules to target tumor cells with specific antigens (Fig. 4). Although promising, particularly for refractory lymphoblastic lymphoma and certain acute leukemias, CAR-T therapy for solid malignancies remains in its early stages. Current EMA and FDA approvals for CAR-T therapies in hematological malignancies are based on promising early trial results. However, their widespread use is restricted to specialized centers due to high costs, significant toxicity requiring close monitoring, and associations with severe adverse events, including cardiovascular complications and inflammatory syndromes [35]. Adoptive CAR-T therapy can lead to severe acute and delayed adverse events, primarily associated with compromised immunity and pathogen spread. These events include hemodynamic alterations, cardiovascular complications, and inflammatory lung syndromes, often with high lethality. Cardiovascular toxicity after CAR-T infusion is linked to triggering inflammatory (CRS), resulting in condition like acute respiratory distress syndrome (ARDS), heart failure, and multi-organ failure. Risk factors such as age, coronary artery disease, and renal impairment are used to assess CAR-T related cardiovascular events [83]. Vascular leak syndrome (VLS), left ventricular dysfunction, and myocardial ischemia have been reported, driven by inflammatory cytokines, causing acute systemic inflammation and vascular impairment [13, 19]. The majority of CAR-T cardiovascular adverse events stem from a massive release of inflammatory cytokines like IL-6, TNF-α and IFN-γ. These cytokines activate the prostaglandin pathway, causing systemic acute inflammatory syndrome with symptoms like fever, tachycardia, hypotension, multi-organ dysfunction, and cardiac damage. Persistent high levels of IL-6 contribute to chronic inflammation leading to cardiovascular impairments like atherosclerosis, arteria hypertension, heart failure, and myocardial ischemia [65]. Retrospective studies on cancer patients receiving CAR-T therapy showed cardiological events including HF, arrhythmias, ACS, myocardial injury, stroke, prolonged QTc and sudden cardiovascular death [2]. Similarly, another study highlighted a direct correlation between CRS onset and risk of MACE, emphasizing the need for continuous cardiovascular monitoring [45]. Finally, in a retrospective study including 116 patients treated with CD19-directed CAR T-cells, 10% experienced new or worsening cardiomyopathy, with a significant decline in left ventricular ejection fraction occurring after treatment initiation [22]. However, these data should be interpreted with caution. In fact, the study by Lefebvre et al. included de novo cardiac arrhythmias in the definition of MACE, which are not typically included in other studies. Furthermore, these retrospective data often report events from the “early days” of CAR-T therapy, where consequently tocilizumab, an IL-6 inhibitor which significantly reduces the clinical impact of CRS, was not used. Additionally, patients treated with CAR T cells are heavily pretreated, which could affect their prognosis. Finally, prospective data reported cardiac decompensation and atrial fibrillation episodes but did not show MACE (defined as a composite of myocardial infarction, stroke, revascularization, ventricular arrhythmias, and arrhythmias requiring implantable devices), contrasting with previous retrospective studies [39]. These cardiovascular events are not exclusive to adults; pediatric patients also showed CRS episodes, left ventricular dysfunction, and hypotension needing vaso-active agents [74]. On these bases, effectively monitoring, diagnosing, and treating CAR-T related cardiotoxicity remains challenging.

Immune effector cell–associated neurotoxicity syndrome (ICANS) should be strictly monitored for their potential ability to sustain the general risk of cardiovascular dysfunctions. Although no data from randomized clinical trials suggest that early intervention reduces the risk of CAR T-associated cardiovascular morbidity, indirect evidence suggest that cardiovascular protection may be achieved when early treatment of CRS takes place with corticosteroids and anti-IL-6 mAbs (tocilizumab) [42].

## Immune-related cardiovascular events

### ICI-related myocarditis and pericarditis

Table 2 summarizes all immune-related cardiovascular side effects with the relative treatment strategies [26, 50]. Myocarditis and pericarditis deserve a special mention since they are the most frequent and severe cardiac irAEs. Pericarditis, myocarditis and myopericarditis are severe immune-related complications, mandating immediate ICI discontinuation, anti-inflammatory therapies and potential pericardiocentesis [41]. The onset of ICI IR-myocarditis generally occurs within 3 months of treatment initiation (80% of cases), though symptoms can appear a few days to years later [11]. Clinical phenotypes of this condition range from smoldering (subclinical) to severe cases presenting with heart failure, arrhythmias or cardiogenic shock [28]. Moreover, cases of myocarditis with myositis and/or myasthenia gravis overlap syndrome (IM3OS) have been reported, with significant mortality and morbidity [61]. The pathogenic mechanisms of ICI-related CTR-CVT are multifaceted. According to Axelrod et al., myocardial immune infiltrate in ICI-related myocarditis is characterized by clonally expanded CD8 + T cells. Depleting these cells in mice significantly improved survival, showing that CD8 + cells are essential for the development of myocarditis. The last-mentioned study identified α-myosin, a heart-specific protein, as the main autoantigen recognized by CD8 + cells [5]. Furthermore, Michel et al. evaluated the direct effects of ICIs on cardiac cells, showing that these drugs can induce cardiomyocyte apoptosis and necrosis through immune-mediated mechanisms. They identified specific pathways, including endothelial dysfunction and increased expression of pro-apoptotic factors, which contribute to cardiotoxicity. Finally, they found that cardiolipins, component of the inner mitochondrial membrane, were significantly higher in anti-PD-1 treated mice, identifying mitochondrial damage and dysfunction as additional pathogenetic pathway in ICI-related CTR-CVT. This mitochondrial impairment affects energy production and increases oxidative stress [53]. Diagnosis should be pursued promptly upon symptom presentation using ECG monitoring, serial troponin and natriuretic peptide (BNP) assays and, progressively, echocardiography, cardiac imaging (cMRI and/or Cardio CT scan) and endomyocardial biopsy if highly suspected. Data indicate that 89% of positive cases present recognizable ECG abnormalities [51]. Elevated troponin (94%) and BNP (66%) levels are reliable early markers of ICI-related myocarditis and increased risk of Major Adverse Cardiac Events (MACE). Echocardiography is the first-line imaging modality, though left ventricular ejection fraction (LVEF) anomalies are undetectable in 50% of cases at the onset [50]. Diagnosis may be confirmed by detecting high troponin and BNP levels alongside LVEF decline and decreased global longitudinal strain (GLS) [4]. Cardiac magnetic resonance imaging (cMRI) has limited sensitivity in ICI-associated myocarditis as reported in an international registry of ICI-associated myocarditis especially if performed at the beginning of the symptoms [24, 94]. Recently, fibroblast activation protein inhibitor (FAPI) PET/CT has been proposed for early detection [21]. Endomyocardial biopsy, while being the definitive diagnostic standard, is invasive nature and risky, limiting its use in cancer patients. Treatment guidelines recommend early administration of corticosteroids, supportive therapy, and permanent interruption of the immune-oncological treatment. Rapid severe onset may necessitate plasmapheresis to remove the excess of ICI mAbs from the bloodstream, timed-according to the half-life of the specific drugs (e.g., ipilimumab 14.5 days, pembrolizumab 25.0 days, nivolumab 26.7 days and atezolizumab 27.0 days) [27]. Severe cases may require immunosuppressant like abatacept [14]. Inpatient management is recommended, including hemodynamic and arrhythmia monitoring. Advanced mechanical circulatory support, including intra-aortic balloon pump, Impella (Abiomed, Danvers, Massachusetts, USA) or extracorporeal membrane oxygenation, may also be required in case of cardiogenic shock [58]. Pericarditis, myopericarditis and pericardial effusion are further immune-related adverse events, mainly occurring in NSCLC patients receiving PD-1/PD-L1 mAbs alone or in combination. Clinical onset includes chest pain and dyspnea with pericarditis-related ECG changes (low voltages and tachycardia) and pericardial effusion on imaging. These immuno-related complications have a high rate of lethality (21%), higher than other pericarditis forms [17]. Treatment requires immediate ICI therapy discontinuation, anti-inflammatories drugs, steroids, supportive therapy, and eventually pericardiocentesis. Proactive cardiac care with close cardiovascular monitoring and prompt therapeutic intervention is essential for cancer patients receiving ICI mAbs, whether alone or in combination.

**Table 2:** Immuno-related cardiovascular events with treatment strategies, adapted from ESMO guidelines [26]

Cardiotoxicity	Clinical presentation	Biomarkers and Instrumental Diagnosis	ICI strategy	Immunosuppression	Cardiac treatment
Myocarditis	Chest pain Dyspnea Pulmonary edema Cardiogenic shock	Troponin NT-proBNP ECG Echocardiography CMR imaging (modified Lake Louis criteria) FAPI-PET-CT Endomyocardial biopsy (Multifocal inflammatory cell infiltrates with overt cardiomyocyte loss by light microscopy) Coronary angiography (exclude ACS)	Stop ICI	First-line: i.v. methylprednisolone 500–1000 mg daily for 3 days or until clinically stable, Follow with oral prednisolone 1 mg/kg o.d. with tapering schedule of 10 mg/week with troponin monitoring High-risk myocarditis: abatacept and ruxolitinib	i.v. diuretics ± nitrates if pulmonary edema ACE inhibitors, Beta blockers
Pericarditis complicated by cardiac tamponade	Chest pain Dyspnea Cardiogenic shock	ECG Echocardiography CMR imaging (for concomitant myocarditis)	Interrupt ICI Consider ICI re-administration when stable and no evidence of ongoing pericarditis	Colchicine 500 μg b.i.d i.v. methylprednisolone 500–1000 mg daily until clinically stable, follow with oral prednisolone 1 mg/kg o.d. with tapering 10 mg/week	Emergency pericardiocentesis Colchicine
Acute pericarditis (with or without effusion but without cardiac tamponade)	Chest pain Dyspnea	ECG Echocardiography CMR imaging (for concomitant myocarditis)	Interrupt ICI Consider ICI re-administration when stable and no evidence of ongoing pericarditis	Colchicine 500 μg b.i.d. and oral prednisolone 0.5 mg/kg o.d. with tapering 10 mg/week	
New advanced conduction disease (second- or third- degree heart block)	Syncope Lipothymia	ECG Holter ECG Serum electrolytes count	Multidisciplinary approach for optimal management of immunological treatment	Consider i.v. methylprednisolone if progressive PR prolongation or any evidence of co-existing myocarditis e.g. elevated troponin, CMR evidence	Emergency pacing
Acute MI	Chest pain Dyspnea Pulmonary edema Cardiogenic shock	Troponin NT pro-BNP ECG Echocardiography CMR Coronary angiography	Multidisciplinary approach for optimal management of immunological treatment	Consider i.v. methylprednisolone if evidence of coronary vasculitis on angiography	Follow ESC/ACC/AHA guidelines for STEMI or NSTEMI Consider vasculitis if atherosclerosis is excluded by coronary angiography
New onset AF	Palpitations Dyspnea Weakness Asymptomatic	ECG Holter ECG Exclude myocarditis	Multidisciplinary approach for optimal management of immunological treatment		Follow ESC guideline for AF Anticoagulation unless contraindication or limited life expectancy
VT or VF	Syncope Hypotension Palpitations Cardiac arrest	ECG Holter ECG	Multidisciplinary approach for optimal management of immunological treatment	First-line: i.v. methylprednisolone 500–1000 mg daily if myocarditis evident until clinically stable and troponin-negative followed by oral prednisolone 1 mg/kg o.d. with tapering 10 mg/week	Emergency defibrillation Beta blockers and/or antiarrhythmics
Takotsubo syndrome	Chest pain Dyspnea Pulmonary edema Cardiogenic shock	Troponin NT pro-BNP ECG Echocardiography CMR ± biopsy Exclusion of ACS according to AHA and ESC guidelines	Multidisciplinary approach for optimal management of immunological treatment		HFA position statement management algorithm
New early conduction abnormality on ECG	Asymptomatic Palpitations Weakness	Troponin NT pro-BNP ECG Holter ECG	Multidisciplinary approach for optimal management of immunological treatment		If high-grade heart block excluded, monitoring with ECG before each cycle
New asymptomatic rise in BNP or NT-proBNP	Asymptomatic	BNP or NT-pro-BNP Troponin ECG Echocardiogram CMR if suspected myocarditis	Multidisciplinary approach for optimal management of immunological treatment		Periodic monitoring Myocarditis treatment if diagnosed
New onset Left Ventricle Systolic Dysfunction (LVSD)	Asymptomatic Dyspnea Pulmonary edema Cardiogenic shock	BNP or NT-pro-BNP Troponin ECG Echocardiogram	Multidisciplinary approach for optimal management of immunological treatment		AHA/ACC/ESC guidelines for heart failure

### Future perspective

Immunotherapy, especially involving ICIs and mAbs, is revolutionizing cancer treatment, expanding its use in managing various malignances. Recently, ICIs have been extended to adjuvant and neoadjuvant settings for early-stage disease, potentially aiding radical surgery and emphasizing the imperative of long-term quality of life and adverse event prevention. Despite fast-track approvals, the comprehensive understanding of ICIs’ short and long-term toxicity and impact on patient’ quality of life remains unclear [40]. Several approaches have been identified to mitigate the cardiotoxic effects of ICIs. Recent evidence supports the use of IL-6 inhibitors, such as tocilizumab, which have shown efficacy in reducing CRS severity without affecting CAR-T cell efficacy [42]. Ruxolitinib, a JAK1/2 inhibitor, is another promising agent in ICI-related CTR-CVT. By inhibiting the JAK-STAT pathway, ruxolitinib mitigates inflammation, thereby reducing the risk of cardiotoxicity. In a recent study of patients with ICI-related myocarditis, treatment with abatacept and ruxolitinib significantly reduced the incidence of MACE [70]. Co-therapies that combine ICIs with cardio-protective agents are another promising approach.

Some irAEs significantly impact the cardiovascular system, affecting patient outcomes, treatment discontinuation, and overall survival in immune-oncological therapies [23]. Approximately, one-third of new cancer cases annually warrant complex immune-oncological treatments, escalating the risk of irAEs. This knowledge acquires a dramatic meaning while considering that a third of the 1.8 million of the new cancer cases per year will have indication for complex immuno-oncological treatments with high risk of irAEs. This review provides detailed, updated evidence on current immunotherapy drugs, including cancer vaccines, highlighting how their side effects differ profoundly from standard chemotherapy in both etiopathogenesis and risk factors. Given these substantial differences, it is essential to conduct specific patient risk assessment based on immunotherapy treatments. In the future, the development of validated scores could help to assess the risk of cardiac complications related to immune-oncological treatments.

## Conclusions

Given the high risk of cardiovascular complications with immuno-oncological treatments, their detrimental impact on patients’ lives, and the lack of recognized clinical or laboratory biomarkers able to predict the risk of IR-cardiovascular events, it is crucial to support the use of these new anticancer drugs with a structurally organized multidisciplinary team. The increased use of immunotherapy and immune checkpoint blockade as standard cancer treatments introduces new challenges in managing cardiovascular immune-related adverse events. This necessitates a new line of research and a tailored approach focused on preventing and treating these severe and potentially lethal complications.

## Data Availability

The data underlying this article will be shared on reasonable request to the corresponding author.
